# Supplementary material for: The Evolution and Characterization of the RNA Interference Pathways in Lophotrochozoa
Source: Genome Biol Evol. 2024 May 7;16(5):evae098. doi: 10.1093/gbe/evae098 (PMC11114477; doi:10.1093/gbe/evae098)
Supplement: evae098_Supplementary_Data [file evae098_supplementary_data.zip › Supplementary_materials.zip!/selected_miRNAs/NoGe_selected_miRNAs/known_miRNA_families_pdf_files/NMRB01000152.1_Notospermus_geniculatus_strain_Ushimado_scaffold152,_whole_genome_shotgun_sequence_5844.pdf]

Provisional ID	:	NMRB01000152.1_Notospermus_geniculatus_strain_Ushimado_scaffold152,_whole_genome_shotgun_sequence_5844
Score total	:	92455
Score for star read(s)	:	3.9
Score for read counts	:	92446.2
Score for mfe	:	1.9
Score for randfold	:	0
Score for cons. seed	:	3
Total read count	:	181341
Mature read count	:	181247
Loop read count	:	0
Star read count	:	94

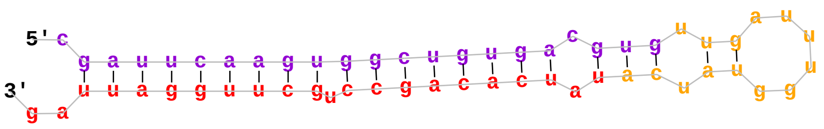

Genome coordinates: NMRB01000152.1 417241-417299

pre-miRNA sequence:
CGAUUCAAGUGGCUGUGACGUGCGAUUCAAGUGGCUGUGACGUGUUGAUUUGGUAUCAUAUCACAGCCUGCUUGGAUUAG

Mature sequence: UAUCACAGCCUGCUUGGAUUAG

Aligned mature sequence of the *N. geniculatus* pre-miRNA with the top 5 hits from the BLAST search against the pre-miRNA mirGeneDB set:

```
                               *************** *** **
Nge_Mir-2-o73                  uaucacagccugcuuggauuag--
Cgi-Mir-2-o29_3p               uaucacagccugcuuggaucagua
Lgi-Mir-2-o16_3p               uaucacagccugcuuggaucagc-
Npo-Mir-2-o24_3p               uaucacagccugcuuugaugaga-
Lgi-Mir-2-o14_3p               uaucacagccugcuuggaucagu-
Lan-Mir-2-o73_3p               uaucacagccugcuuugaugaga-
```
